# Accurate, Streamlined Analysis of mRNA Translation by Sucrose Gradient Fractionation

**DOI:** 10.21769/BioProtoc.2573

**Published:** 2017-10-05

**Authors:** Soufiane Aboulhouda, Rachael Di Santo, Gabriel Therizols, David Weinberg

**Affiliations:** 1 Department of Molecular and Cellular Pharmacology, University of California, San Francisco, CA, USA; 2 Sandler Faculty Fellows Program, University of California, San Francisco, CA, USA

**Keywords:** Translation, Gene regulation, Ribosome, Polysome analysis, Sucrose gradient fractionation, Reproducibility

## Abstract

The efficiency with which proteins are produced from mRNA molecules can vary widely across transcripts, cell types, and cellular states. Methods that accurately assay the translational efficiency of mRNAs are critical to gaining a mechanistic understanding of post-transcriptional gene regulation. One way to measure translational efficiency is to determine the number of ribosomes associated with an mRNA molecule, normalized to the length of the coding sequence. The primary method for this analysis of individual mRNAs is sucrose gradient fractionation, which physically separates mRNAs based on the number of bound ribosomes. Here, we describe a streamlined protocol for accurate analysis of mRNA association with ribosomes. Compared to previous protocols, our method incorporates internal controls and improved buffer conditions that together reduce artifacts caused by non-specific mRNA–ribosome interactions. Moreover, our direct-from-fraction qRT-PCR protocol eliminates the need for RNA purification from gradient fractions, which greatly reduces the amount of hands-on time required and facilitates parallel analysis of multiple conditions or gene targets. Additionally, no phenol waste is generated during the procedure. We initially developed the protocol to investigate the translationally repressed state of the *HAC1* mRNA in *S. cerevisiae*, but we also detail adapted procedures for mammalian cell lines and tissues.

## Background

The translation of mRNA into protein is a highly regulated process that can occur at different rates depending on the gene, cellular context, or environment. Each step of translation–initiation, elongation, and termination–can be a point of regulation that ultimately affects the number of ribosomes associated with an mRNA (Dever and Green, 2012; Hinnebusch and Lorsch, 2012). Because the time between consecutive initiation events is usually shorter than the time required for elongation, most mRNAs are associated with more than one ribosome at a time to form ‘polysome’ structures (Warner *et al*., 1963). Thus, the ability to count the number of ribosomes per mRNA molecule provides an assay for the overall translation state of mRNA. Traditionally, this counting has been achieved by sucrose gradient fractionation (also sometimes called polysome analysis), in which mRNAs are separated by ultracentrifugation based on their size/shape and then quantified (Mašek *et al*., 2011). The detection of mRNAs in gradient fractions can either be done for individual mRNAs by RNA blotting or qRT-PCR, or for the entire transcriptome by microarrays or high-throughput RNA sequencing (Arava *et al*., 2003; Floor and Doudna, 2016). In this way, the absolute number of ribosomes associated with individual mRNA molecules can be determined. An alternative method for assaying translation is ribosome-footprint profiling, in which short fragments of mRNA that are protected from RNase digestion by ribosomes are captured and subjected to high-throughput sequencing (Ingolia *et al*., 2009). When combined with total RNA sequencing to determine mRNA abundances, ribosome-profiling data can measure the translational efficiencies of mRNAs in a genome-wide manner. However, ribosome profiling provides only a relative measure of translational efficiency that can be biased by RNA-abundance measurements (Weinberg *et al*., 2016). In addition, ribosome profiling is not well suited to the study of low-abundance mRNAs or when only a small number of mRNAs are of interest. For these reasons, sucrose gradient fractionation remains an important tool for the analysis of translational efficiency.

We present an adaption of this widely used technique that incorporates key features that improve accuracy and reduce hands-on time. mRNA polysome analysis by sucrose gradient fractionation is completed in three steps: lysate preparation, sucrose gradient fractionation, and RNA-abundance analysis. Our protocol was initially developed to streamline the analysis of multiple RNAs in parallel, but in the process of protocol development we also carefully optimized each step to ensure that the assay provided an accurate and reproducible measure of ribosome association. We developed the protocol for the budding yeast *S. cerevisiae* (Di Santo *et al*., 2016) but since then have also applied it to a wide variety of human and mouse cell lines and even whole mouse tissues (Odegaard *et al*., 2016). A key feature of our protocol is the inclusion of heparin in the lysis buffer, which reduces non-specific interactions between mRNA and ribosomes that can otherwise lead to artefactual co-sedimentation of untranslated mRNAs with polysomes. We also incorporate a reliable control for untranslated RNA: an un-capped exogenous RNA that is spiked into the lysate prior to ultracentrifugation. For the RNA analysis step we adapted a qRT-PCR kit previously used for cell lysates to work directly with gradient fractions, thus eliminating the time-consuming RNA purification steps used in all previous polysome analysis protocols. Measuring RNA abundances directly from crude gradient fractions not only reduces time requirements and hands-on manipulations but also eliminates generation of phenol waste. Finally, to control for variations in RT-PCR efficiencies among fractions (which differ in sucrose concentration and macromolecular composition), we spike in an equal amount of artificial RNA to each fraction just after collection to serve as a normalization reference. In summary, our protocol–presented in detail below–contains a collection of improvements and internal controls that together provide an accurate, streamlined assay for polysome analysis.

## Materials and Reagents

### A. STAGE 1: Lysate preparation

#### Materials

##### Yeast

1. Inoculation loop (Fisher Scientific, catalog number: 22-363-604)
2. 50 ml conical tube (Corning, Falcon^®^, catalog number: 352098)
3. 1.5 ml siliconized G-tube (Bio Plas, catalog number: 4165SL)
4. 0.45 micron filters (Pall, catalog number: 60206)
5. Cell lifter (Corning, catalog number: 3008)

##### Mammalian cells

1. 6-well plate (Corning, catalog number: 3516) or 10-cm cell culture dish (Corning, catalog number: 353803)
2. Cell lifter (Corning, catalog number: 3008)
3. 15 ml conical tube (Corning, Falcon^®^, catalog number: 352096)
4. 1.5 ml siliconized G-tube (Bio Plas, catalog number: 4165SL)

##### Tissues

1. 50 ml conical tubes (Corning, Falcon^®^, catalog number: 352098)
2. 5 ml centrifuge tubes (Eppendorf, catalog number: 0030119401)
3. 1.5 ml siliconized G-tubes (Bio Plas, catalog number: 4165SL)

#### Reagents

1. Liquid nitrogen (LN_2_)
2. Appropriate culturing media (*e.g*., YPD for *S. cerevisiae*; DMEM, FBS and additives for mammalian cell lines)
3. 1x phosphate-buffered saline (PBS) (Thermo Fisher Scientific, Gibco™, catalog number: 10010023)
4. Luciferase RNA (Promega, catalog number: L4561)
  Note: Store aliquotted 100 ng/μl stock at −80 °C.
5. HEPES (Sigma-Aldrich, catalog number: H4034)
6. Magnesium chloride (MgCl_2_) (Sigma-Aldrich, catalog number: 68475)
7. Potassium chloride (KCl) (Sigma-Aldrich, catalog number: P9541)
8. Heparin (Sigma-Aldrich, catalog number: H3149)
  Note: Store 10 mg/ml stock solution at 4 °C.
9. Triton X-100 solution (Sigma-Aldrich, catalog number: 93443)
10. Dithiothreitol (DTT) (Sigma-Aldrich, catalog number: D9779)
  Note: Store filtered and aliquotted 1 M stock solution at −20 °C.
11. Cycloheximide (AMRESCO, catalog number: 94271)
12. Superase-IN (Thermo Fisher Scientific, Invitrogen™, catalog number: AM2696)
  Note: Store filtered and aliquotted 100 mg/ml stock solution at −20 °C.
13. cOmplete, mini, EDTA-free Protease Inhibitor Cocktail (Roche Diagnostics, catalog number: 11836170001)
14. Sucrose (Sigma-Aldrich, catalog number: S5016)
  Note: Store solution at 4 °C, see Recipes section.
15. Lysis buffer (see Recipes)
16. 10% sucrose solution (see Recipes)
17. 50% sucrose solution (see Recipes)

### B. STAGE 2: Sucrose gradient fractionation

#### Materials

1. 50 ml SteriFlip (EMD Millipore, catalog number: SCGP00525)
2. Open top polyclear centrifuge tubes (Seton Scientific, catalog number: 7030)
3. SW41 marker block (included with fractionator)
4. 60 ml syringe (BD, catalog number: 309653)
5. Stainless steel syringe needle, noncoring point, ~10 inches, ~12 gauge (Sigma-Aldrich, Cadence Science, catalog number: Z116971)
6. Short caps (Biocomp, catalog number: 105-514-6)
7. Tube rack (Beckman Coulter, catalog number: 331313)
8. 2 ml tubes w/screw caps (USA Scientific, catalog number: 1420-8700)
9. Cling film or Parafilm

### C. STAGE 3: mRNA analysis

#### Materials

1. qPCR plates (RPI, catalog number: 141328)
2. qPCR film (Bio-Rad Laboratories, catalog number: MSB1001)
3. PCR tubes

#### Reagents

1. Cells-to-Ct kit (Thermo Fisher Scientific, Invitrogen™, catalog number: AM1728)
2. Superase-In (Thermo Fisher Scientific, Invitrogen™, catalog number: AM2696)
3. XenoRNA (Thermo Fisher Scientific, Invitrogen™, catalog number: 4386995, part of control kit)
  Note: Store in small aliquots at −80 °C.
4. TaqMan Gene Expression Master mix (Thermo Fisher Scientific, Applied Biosystems™, catalog number: 4369016)
5. Primer/Probe qPCR assays for genes of interest (Thermo Fisher Scientific, Ac00010014_a1 (XenoRNA) and Mr03987587_mr (Luciferase))

## Equipment

1. Pipette (*e.g.*, P1000, P10)
2. 250 ml baffled flask (Corning, PYREX^®^, catalog number: 4444-250)
3. 2 L baffled flask (Corning, PYREX^®^, catalog number: 4444-2L)
4. Filtration system (Restek, catalog number: KT676001-4035)
5. Coors porcelain mortar and pestle (Sigma-Aldrich, catalog numbers: Z247472 and Z247510) *Manufacturer: CoorsTek, catalog numbers: 60316 and 60317.*
6. Dounce, tissue grinder (DWK Life Sciences, WHEATON, catalog number: 357538) [optional]
7. Tabletop cold centrifuge (Eppendorf, model: 5424 R)
8. SW41 Ti rotor (Beckman Coulter, model: SW 41 Ti, catalog number: 331362)
9. Ultracentrifuge (Beckman Coulter, model: L8-80M)
10. Gradient station (Biocomp, catalog number: 153-001)
11. Fraction collector (Gilson, catalog number: FC 203B)
12. BIORAD EM-1 Econo UV monitor (Bio-Rad Laboratories, model: EM-1 Econo™)
13. NanoDrop 2000 spectrophotometer (Thermo Fisher Scientific, Thermo Scientific, model: NanoDrop™ 2000, catalog number: ND-2000)
14. CFX96 Touch Real-Time PCR detection system (Bio-Rad Laboratories, model: CFX96 Touch™, catalog number: 1855195)

## Software

1. Gradient Profiler V2’ software

## Procedure

**Figure 1:**
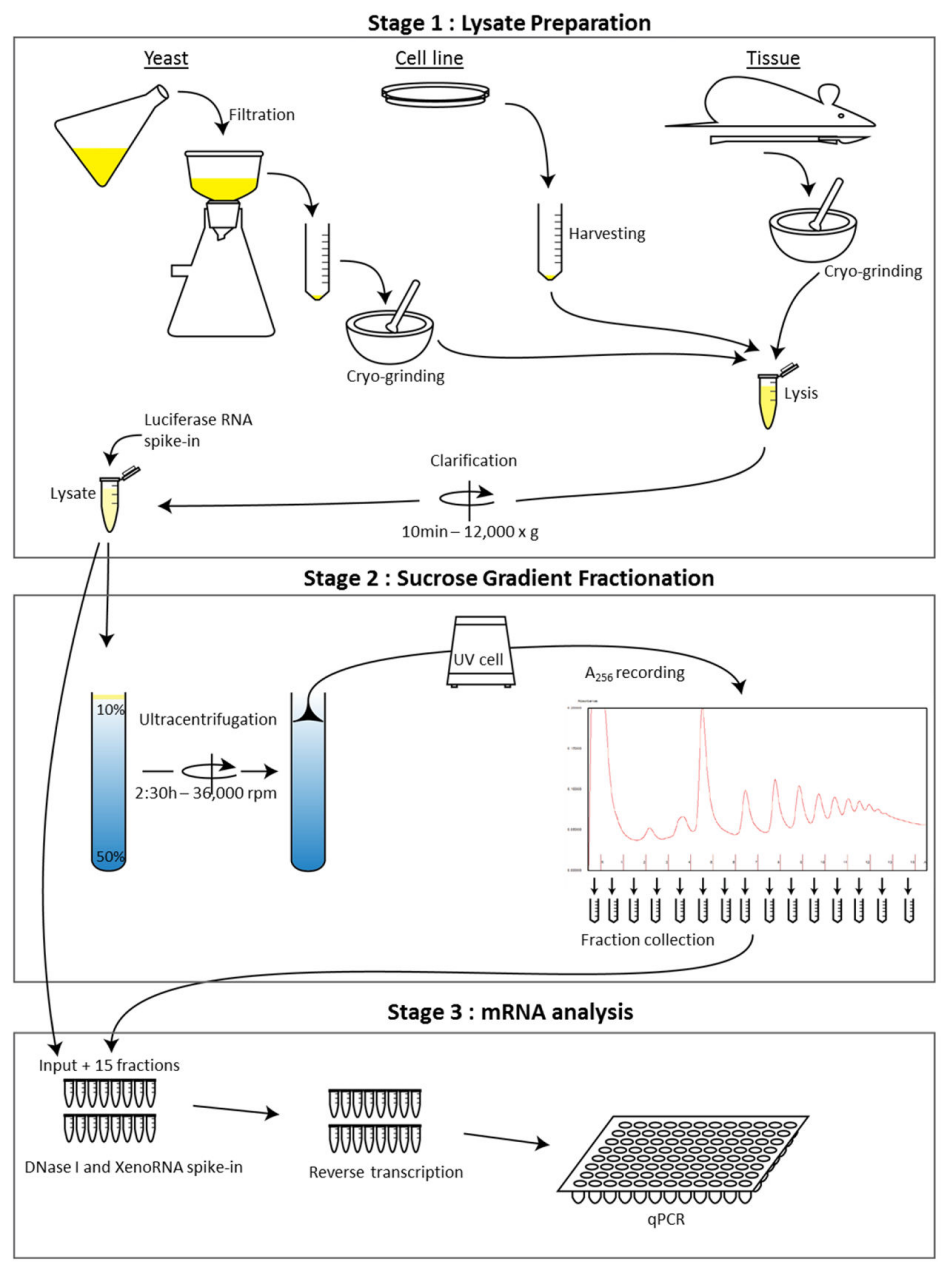
Workflow schematic of the sucrose polysome gradient protocol from multiple cell types This illustrates the overall steps of the procedure to analyze RNA distribution across a polysome gradient.

The protocol is divided into three stages (see Figure 1):

**Stage 1 Lysate Preparation**
  1A. Growth and harvesting of cells (a. yeast, b. cells lines, c. tissues)
  1B. Sample preparation
**Stage 2 Sucrose Gradient Fractionation**
  2A. Gradient preparation
  2B. Ultracentrifugation
  2C. Fractionation
**Stage 3 mRNA Analysis**
  3A. DNase treatment and control RNA spike-in
  3B. Reverse transcription
  3C. Real-time PCR

The recommended workflow timing is:


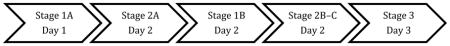


Note: Stage 2A requires an incubation of 30–60 min and is carried out prior to Stage 1B to optimize timing.

### A. STAGE 1: Lysate Preparation

#### 1. PART 1A. Growth and harvesting of cells

##### Yeast

- Day 0
  - Inoculate cells from plate into 50 ml YPD in a 250 ml baffled flask.
  - Grow overnight to saturation.
- Day 1
  - Dilute overnight culture into 400 ml YPD at an OD_600_ of 0.05 in a 2 L baffled flask.
    Note: If growing multiple cultures, stagger culture growth slightly (30 min difference between first and last cultures is sufficient).
  - When culture reaches an OD_600_ of 0.5–0.6, harvest cells by rapid filtration:
    1. Pour YPD on filter prior to pouring culture.
    2. Pour entire culture down the side of the filtration vessel, taking care to avoid pouring foam that will collect on top of the filter.
    3. As the last liquid pours through, quickly remove the clamp and top of the filter unit, scrape cells from the filter quickly but gently using a cell lifter, and submerge into conical containing liquid nitrogen. The total time between the last liquid flowing through the filter and the cells being submerged in liquid nitrogen should not exceed 5 sec.
      Note: Cell scraper should be pre-chilled in liquid nitrogen.
  - Place conical with pellet in a −80 °C freezer and allow liquid nitrogen to boil off.
    Note: Leave cap loosely tightened.
  - Lyse cells by grinding with a mortar and pestle.
    1. Pre-chill mortar and pestle with liquid nitrogen (~2–3 min) in an ice bucket.
    2. Pour out any residual LN_2_ from the mortar.
    3. Add the cell pellet to the mortar.
    4. Gently pour ~1–5 ml of LN_2_ on the cell pellet.
      Note: Adding too much LN_2_ will significantly increase processing times.
    5. Grind with a pestle to break apart cells until all LN_2_ boils off, then grind the dry powder for an additional ~1–2 min.
      Note: After evaporation of all the liquid nitrogen in the mortar, the pellet reaches a powder-like consistency quickly, 1–2 min. No benefits are gained from further grinding. From experience, there are no adverse effects of grinding for too long.
    6. Re-suspend the cell powder in liquid nitrogen and carefully pour back into the original conical tube.
  - Place the conical tube in a −80 °C freezer and allow liquid nitrogen to boil off.
    Note: Leave cap loosely tightened.
  - Pause point.
  - Proceed to Stage 2A.

##### Mammalian cells

- Day 0
  - Plate cells as required by experiment.
    Note: The procedure has been successfully applied to various mammalian cell lines cultured in 6-well plates, 10-cm dishes, and 15-cm dishes, with a harvested range of 10^6^ to 10^7^ cells. Confluency at time of harvesting should be avoided by controlling plating density it is important to consider the effects of cell manipulation on translation. Over-confluence, depleted nutrients or serum, or media changes can induce quick translational responses. Using a stable cell line is recommended over transiently transfected cells to ensure reproducibility.
  - Incubate cells under optimal growth conditions.
- Day 1
  - Add cycloheximide (CHX) to media at a final concentration of 100 μg/ml, incubate for 10 min at 37 °C. This step can be omitted.
    Note: While CHX pre-treatment in growth media is optional, we recommend adding CHX to the PBS and lysis buffer to prevent ribosome run-off during harvesting.
  - Pre-chill PBS and lysis buffer on ice and add additives (see Recipes).
  - Transfer tissue culture dish to an ice bucket.
  - Aspirate media.
  - Wash the dish twice with 10 ml ice-cold PBS.
  - Scrape cells thoroughly and quickly in 5 ml of ice-cold PBS.
  - Transfer cell suspension to a 15 ml conical tube.
  - Centrifuge for 5 min at 4 °C at 500 *x g*, discard supernatant.
  - Flash freeze cell pellet and store at −80 °C.
  - Proceed to Stage 2A.

##### Mammalian tissue

- Day 0
  - Dissect out a whole tissue sample.
  - Wash tissue with ice-cold PBS prior to freezing in liquid nitrogen.
- Day 1
  - Break apart and lyse tissue by grinding with a mortar and pestle.
    1. Pre-chill mortar and pestle with liquid nitrogen (~2–3 min) in an ice bucket.
    2. Pour out any residual LN_2_ from the mortar.
    3. Add the frozen tissue to the mortar.
    4. Pour ~1–5 ml of LN_2_ on the frozen tissue.
      Note: Adding too much LN_2_ will significantly increase processing times.
    5. Grind with a pestle to break apart cells until all LN_2_ boils off, then grind the dry powder for an additional ~1–2 min. *Note: After evaporation of all the liquid nitrogen in the mortar, the pellet reaches a powder-like consistency quickly, 1–2 min. No benefits are gained from further grinding. From experience, there are no adverse effects of grinding for too long.*
    6. Re-suspend the cell powder in liquid nitrogen and pour back into the original conical tube.
  - Place the conical tube in a −80 °C freezer and allow liquid nitrogen to boil off.
    Note: Leave cap loosely tightened.
  - Pause point.
  - Proceed to Stage 2A.

#### 2. PART 1B: Sample preparation (Day 2)

Note: ALL following steps are done on an ice block or in a 4 °C cold room.

##### Yeast procedure

- Thaw grinded powder on ice for 5 min. Prematurely adding lysis buffer can cause it to freeze.
- In the meantime, pre-label four siliconized microcentrifuge tubes per sample:
  Re-suspended powder
  Clarified undiluted lysate
  Clarified diluted lysate
  Aliquoted lysate (multiple tubes)
    Note: You will also need three 0.6 ml tubes per sample containing 90 μl ddH_2_O.
- Add 1 ml lysis buffer to the cell powder in each conical.
- Swirl each tube to lightly mix, then fully re-suspend by pipetting up and down using a P1000.
- Transfer entire tube contents to pre-labeled ‘re-suspended’ 1.7 ml tubes.
- Spin for 10 min at 1,300 *x g* at 4 °C.
- Transfer clarified lysate (~800 μl) into ‘clarified undiluted’ labeled 1.7 ml tubes. Clarified lysate should have a translucent appearance with a white/yellow hue.
- Transfer 10 μl into 90 μl water to spec on NanoDrop for RNA concentration, which serves as a proxy for total lysate concentration.
  Notes:
  - Triton X-100 interferes with reading so dilution is needed.
  - Blank will have 10 μl lysis buffer + 90 μl ddH_2_O.
- Dilute all lysates to 25 OD_260_ U/ml (1 μg/μl RNA) with lysis buffer.
- Spec the diluted lysate to ensure that all samples are within ~5% of each other.
- Add exogenous uncapped Luciferase RNA (Promega) to a final concentration of 100 ng/ml.
- Aliquot 150 μl into 1.7 ml tubes.
- Store lysates not immediately needed for experiment at −80 °C.
- Proceed to Stage 2B.

##### Mammalian cells procedure

- Thaw cell pellet on ice.
- Resuspend cell pellet in 100 μl lysis buffer per 10^6^ cells.
- Transfer lysate to a 1.7 ml tube.
- Incubate for 10 min on ice, mix by pipetting up and down.
  Note: Optimal lysis time and detergent concentration may vary depending on the cell type. Check cell lysis under a microscope with phase contrast at different times during lysis. Triton can be substituted by other detergents such as NP-40 or mechanical lysis using a dounce homogenizer.
- Centrifuge for 10 min at 4 °C at 12,000 *x g*.
- Transfer clarified lysate into a ‘clarified undiluted’ labeled 1.7 ml tube.
- Dilute 10 μl of lysate into 90 μl water to spec on NanoDrop for RNA concentration, which serves as a proxy for total lysate concentration (see Note 8).
- Dilute all samples to the same concentration by adding an appropriate amount of lysis buffer.
  Note: We recommend diluting to ~20–100 μg/ml. Adjust concentrations and measure by spec to ensure all samples are within 5% of each other.
- Spike in exogenous uncapped Luciferase RNA to a final concentration of 100 ng/ml.
- Aliquot 200–500 μl into 1.7 ml tubes and store the remaining lysate (input) at −80 °C.
- Proceed to stage 2B.

##### Tissue procedure

- Weigh 50 mg (one scoop) of frozen powder into LN_2_ chilled 5 ml Eppendorf tubes.
  - Act quickly to avoid tube warming up.
  - Dip tubes into LN_2_ and shake to separate powder frequently (main 50 ml conical).
- Let to ‘thaw’ to 4 °C in ice before adding lysis buffer (LB).
- Add 50–100 μl of lysis buffer per mg of powder.
- Pipette up and down to mix, vortex vigorously, and let sit on ice.
  - Allow Triton X-100 to lyse lipids for 5–10 min after adding LB before spinning.
  - Vortex again.
- Spin 750 *x g* for 10 min at 4 °C.
- Separate supernatant into a new tube.
  Note: Whole tissue samples: Lipid-rich samples must be carefully prepared to avoid lipid contamination. As such, we recommend taking the middle 75% of the clarified lysate after centrifugation to avoid disturbing the top lipid layer or bottom insoluble material.
- Spin 12,000 *x g* for 10 min at 4 °C.
- Separate supernatant into a new tube.
  Note: Take 75% liquid from the middle.
- Transfer 10 μl of supernatant into 90 μl water to spec on NanoDrop for RNA concentration, which serves as a proxy for total lysate concentration (see Note 8).
- Dilute all samples to 100 ng/μl RNA in lysis buffer containing Heparin.
- Add exogenous uncapped Luciferase RNA (Promega) to a final concentration of 100 ng/ml, then vortex to mix.
- Aliquot 250 μl into 1.7 ml tubes and store the remaining lysate (input) at −80 °C.
- Proceed to Stage 2B.

### B. STAGE 2: Sucrose Gradient Fractionation (Day 2)

#### 1. PART 2A. Gradient preparation

The set up should be done at room temperature and prior to the second step of lysate preparation. While gradients are cooling to 4 °C, prepare and clarify the lysates.

- Prepare sucrose solutions
  - Aliquot 40 ml of pre-filtered sucrose solutions (stored at 4 °C, see Recipes section) into a conical tube, and let warm to room temperature.
  - Add DTT, cycloheximide, and Superase-IN to sucrose solutions, then mix by gentle rotation.
- Prepare lysis buffer and put on ice to cool to 4 °C.
- Mark Polyclear centrifuge tubes using the SW41 Ti marker block by drawing a line on each tube at the top marker block line.
- Using a stripette, fill centrifuge tubes with 10% sucrose solution (see Recipes) up to ~2 mm above the marked line.
- Fill up a 50 ml syringe with the 50% sucrose solution (see Recipes) slowly (to avoid bubbles). Attach the cannula and expel any air by holding the syringe vertically (with the cannula pointing up).
- Holding the tube such that the marked line is at eye level, quickly and vertically insert the cannula into the bottom of the tube (avoiding the 50% sucrose solution leaking into the 10% solution).
- Slowly expel the 50% sucrose solution while maintaining the bottom of the cannula ~5 mm below the meniscus. When the meniscus of the interphase layer reaches the marked line, stop expelling and quickly pull out the cannula.
- Cap each tube (taking care to avoid any air pockets).
- Using a P1000, pipette out any residual sucrose that was pushed out through the cap’s hole.
- Place tubes into the gradient maker tube holder (that has been pre-leveled using the manufacturer-supplied level).
- Using the gradient maker station software, run the ‘14S short 10–50%’ program (see Note 1 for program information).
- Transfer the tubes to the cold room (but do not remove caps yet) while you prepare the lysates.
- At this point, turn on the ultra-centrifuge to allow it to pre-cool to 4 °C.

#### 2. PART 2B. Ultracentrifugation

- Gently remove caps from 10–50% sucrose gradients.
- Slide sucrose-gradient tubes into rotor buckets.
- Remove (X + 100) μl from the top of each gradient, where X is the amount of lysate you will load (typically 100 μl but up to 600 μl is acceptable).
  Note: The downstream RNA analysis steps of this protocol work best for sucrose gradients performed using < 100 μg of lysate (based on A_260_ units). For higher loading of lysates some scaling up and optimization of the downstream steps of the protocol may be required.
- Slowly layer 100–600 μl of lysate on top of the gradient. The lysate should form a visible and neat layer.
  Note: Save at least 10 μl of lysate as the ‘Input’ fraction for downstream qRT-PCR analysis.
- Weigh each gradient tube in a balance and carefully adjust the weight of each tube, if needed, by adding lysis buffer. Equilibrate the bucket pairs facing each other on the rotor: 1–4, 2–5 and 3–6.
- Cap the buckets.
- Attach buckets to the SW41 Ti rotor.
- Gently lower the rotor into the centrifuge, and lightly spin the rotor by hand to ensure that all buckets are connected properly.
- Enter centrifugation settings:
  Vacuum–ON
  Temp–4 °C
  Speed–36,000 rpm (160,000 *x g*)
  Time–2.5 h
  Acceleration–1
  De-acceleration–7
- Start the centrifuge and ensure that it reaches the desired speed. The centrifuge may pause acceleration at 3,000 rpm until the vacuum is fully engaged.
  Note: On an SW41 Ti rotor, 36,000 rpm corresponds to 160,000 x g at r_av_. If you are using a different rotor, please refer to the manual to use the correct speed.

#### 3. PART 2C. Fractionation

Read and follow manufacturer’s instructions. We recommend contacting the local Biocomp representative for an advanced tutorial.

- During the spin, turn on the Bio-Rad Econo UV monitor to warm up and label and chill screw-cap tubes.
  Notes:
  - Allow the Bio-Rad Econo UV Monitor to warm up for at least 2 h before setting the zero.
  - During centrifugation: Pre-label 16 screw cap tubes (USA Scientific) per gradient, cover with cling film or Parafilm to prevent dust/RNase contamination and store in at 4 °C.
- Turn on the Gilson fraction collector, the Biocomp gradient station, the computer and open ‘Gradient Profiler V2’ software.
- Set the zero UV reading with clean water Bio-Rad Econo UV monitor.
- Ensure that UV readout is stable, not fluctuating.
- Remove rotor from centrifuge, place rotor tubes on rack, and place in cold room.
  Note: Do not remove screw cap until needed for fractionation.
- Fractionate gradients into 2 ml screw-cap tubes using the following settings:
  Note: If at any point the Econo-UV monitor light turns red, pull up the piston, release the air valve, and repeat the zeroing with water.
Speed:0.30 mm/secTotal distance:75 mmNumber of fractions:15Distance/fraction:5.00 mmVolume/fraction:0.71 ml
- Store fractions in the cold room until the entire set of samples have been fractionated.
- Flash freeze all tubes and store at −80 °C.

### C. STAGE 3: mRNA Analysis (Day 3)

#### 1. PART 3A. DNase treatment and control RNA spike-in

- Thaw fraction tubes, input tubes, and Cells-to-Ct stop solution.
- Dilute the input samples 30-fold by adding 6 μl to 174 μl RNase-free water, then put on ice.
- Prepare a Master mix of lysis solution containing the following (per sample):
9.9 μlCells-to-Ct lysis buffer0.1 μlCells-to-Ct- DNase0.1 μlXenoRNA
- Per gradient, prepare 16 PCR tubes (to be used for 15 fractions plus the input) containing 10.1 μl lysis solution master mix.
- Add 1 μl of each fraction (or input) directly into the lysis master mix (*i.e.*, not to the tube wall), then pipette up and down 2–3 times.
- Invert tubes several times to mix gently, then briefly spin down.
- Incubate at room temperature for 5 min, then put on ice (during this incubation you can put the fraction tubes back into −80 °C freezer).
- Pipet 1 μl Cells-to-Ct stop solution directly into each PCR tube (*i.e*., not to the tube wall).
- Invert tubes several times to mix gently, then briefly spin down.
- Incubate at room temperature for 2 min, then put on ice.

#### 2. PART 3B. Reverse transcription protocol

- Prepare RT Master mix containing the following (per sample):
5 μl2x Cells-to-Ct RT buffer0.5 μl20x Cells-to-Ct RT enzyme mix
- Use P10 to distribute 5.5 μl RT master mix to PCR tubes.
- Use multichannel P10 to add 4.5 μl of lysate.
- Perform RT reaction in a thermocycler with the following program: 37 °C for 1 h, 95 °C for 5 min, 4 °C forever.
- Dilute each RT reaction by adding 50 μl water and mixing thoroughly.
- Store at −20 °C or proceed directly to PCR.

#### 3. PART 3C. Quantitative real-time PCR protocol Every fraction is analyzed with qPCR technical duplicates for each probe

- Program instrument for TaqMan assay:
  - Probes are labeled with FAM dye and nonfluorescent quencher.
  - Cycling conditions: 50 °C for 2 min (UDG incubation), 95 °C for 10 min (enzyme activation), 40x [95 °C for 15 sec + 60 °C for 1 min] (PCR).
- Mix 2x TaqMan Gene Expression Master mix by swirling the bottle, mix 20x assays by vortexing briefly and centrifuging; keep all solutions on ice.
- For each gene-of-interest (including the Xeno and Luciferase controls), prepare a TaqMan PCR Cocktail containing (for each qPCR reaction) 5 μl 2x TaqMan Gene Expression Master MIX + 0.5 μl 20x TaqMan assay (gene specific).
- Use a P10 to distribute 5.5 μl of PCR cocktail into a real-time PCR plate at room temperature.
- Use a multichannel P10 to add 4.5 μl of RT reaction for each qPCR reaction, mix by pipetting.
- Cover the plate carefully and briefly centrifuge (~800 *x g* for a few seconds).
- Place reactions in a real-time PCR instrument and start the run.

## Data analysis

**Figure 2:**
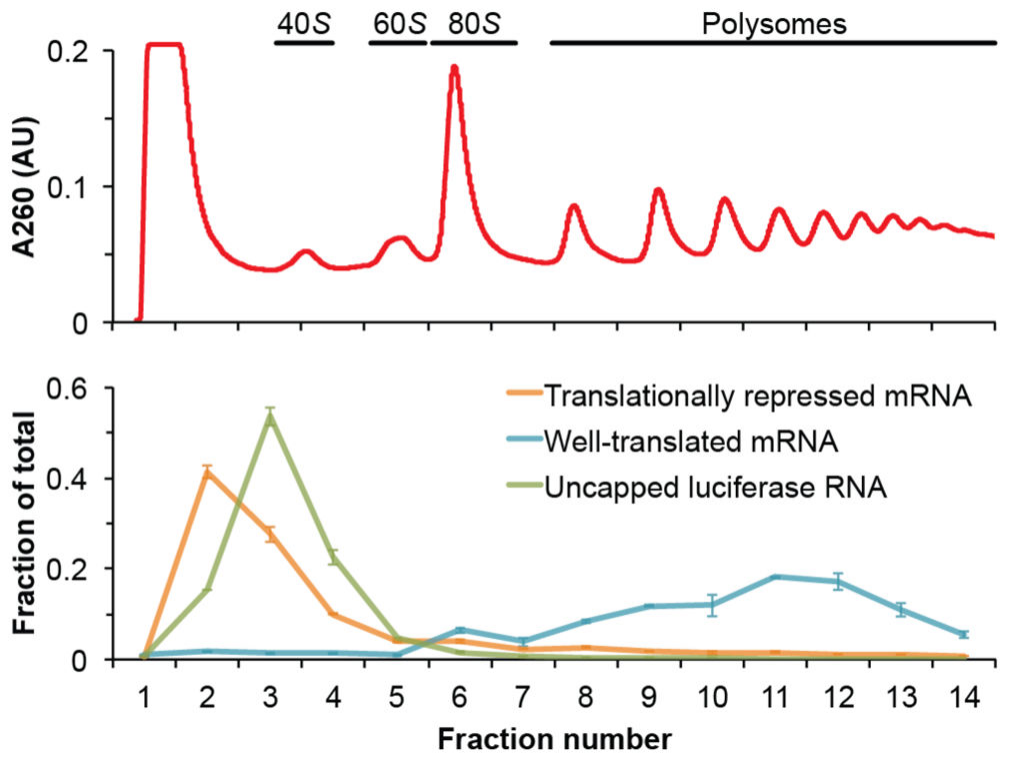
Representative data generated from this polysome gradient analysis protocol The top panel shows the A_260_ absorbance trace of a fractionated yeast lysate with the species of ribosome associated with each peak annotated. The bottom panel shows a representative plot of the relative distribution of RNA associated with each fraction of the gradient as analyzed by qRT-PCR. Represented is a translationally repressed mRNA (orange), a well-translated mRNA (blue) and the uncapped luciferase RNA (green), which serves as a control for non-specific interactions. The well-translated mRNA is mainly polysomic and sediment deep in the gradient toward the bottom of the tube. Both the translationally repressed mRNA and the exogenous control RNA are not associated to ribosomes and remain in the top fractions.

1. Per sample, gather raw Cq information from the qPCR machine for:
  - Xeno
  - Luciferase
  - Actin (or other well-translated gene)
  - Additional genes of interest
2. Assemble values by fraction numerical order.
3. Average Cq values from technical duplicates. Also calculate the difference between replicates and repeat qPCR reactions for any samples a difference greater than 0.5 Cq units (see Note 2).
4. Calculate mRNA abundance in each fraction relative to the input (Pfaffl, 2001) taking into account differences in qRT-PCR efficiency calculated by normalizing to XenoRNA Cq values:
  $$\mathrm{Target}\;\mathrm{abundance}\;in\;\mathrm{fraction}\_X=\frac{2^{Cq_{\mathrm{target},\mathrm{input}}-Cq_{\mathrm{target},\mathrm{fraction}\_X}}}{2^{Cq_{\mathrm{Xeno},\mathrm{input}}-Cq_{\mathrm{Xeno},\mathrm{fraction}\_X}}}$$
5. Convert relative RNA abundances to the percent of total detected RNA:
  $$\mathrm{Percent}\;in\;\mathrm{fraction}\_X=\frac{\mathrm{Target}\;\mathrm{abundance}\;in\;\mathrm{fraciton}\_X}{\sum_{Y=1}^{15}\mathrm{Target}\;\mathrm{abundance}\;in\;\mathrm{fraction}\_Y}$$
6. For each gradient, generate line plots with fraction numbers on the x axis and ‘Percent of total mRNA’ for each target on the y axis.
  *Note: All of the above analysis should be automated in a spreadsheet. In this way, the researcher only needs to copy and paste Cq values to receive all abundance information, quality control metrics, and polysome plots (see Note 2 for troubleshooting). *Figure 2 shows an example of the data analysis with this procedure.

## Notes

1. Fractionation program setup:
  Gradient Master program for 10–50% sucrose gradient:
    - 05/85/35
    - 01/77/0
    - 04/86/35
    - 03/86.5/35
    - 20/81/14
    - 07/86/20
  Sequence of steps: abcbdbabcbdbef
2. Ideally the averaged Cq values for XenoRNA will be roughly the same in all fractions and input (since equal amounts of XenoRNA were added to samples before qRT-PCR). In practice, we allow a range of up to 1 Cq value; a larger range indicates issues with qRT-PCR efficiency in some fractions, which may reflect an overly concentrated or ‘dirty’ lysate. Consider repeating the experiment if > 5% of the uncapped RNA is found associating with polysomes. If a fraction is significantly different in all probe’s Cq values, there was likely a problem introduced at the RT step. If a fraction is significantly different in one probe’s Cq values, there was likely a problem introduced at the qPCR step.

## Recipes

1. Lysis buffer (made fresh each time)
  20 mM HEPES-KOH (pH 7.4)
  5 mM MgCl_2_
  100 mM KCl
  200 μg/ml Heparin
  1% Triton X-100
  2 mM DTT
  100 μg/ml cycloheximide
  20 U/ml Superase-IN
  cOmplete mini EDTA-free Protease Inhibitor Cocktail (1 tablet per 10 ml solution)
2. 10% sucrose solution
  Base: 20 mM HEPES-KOH (pH 7.4), 5 mM MgCl_2_, 100 mM KCl, 10% sucrose
  Filter sterilize. Store at 4 °C for > 2 weeks
  Additives added fresh each time (final concentration): 2 mM DTT, 100 μg/ml cycloheximide, 20
  U/ml Superase-IN
3. 50% sucrose solution
  Base: 20 mM HEPES-KOH (pH 7.4), 5 mM MgCl_2_, 100 mM KCl, 50% sucrose
  Filter sterilize. Store at 4 °C for > 2 weeks
  Additives added fresh each time (final concentration): 2 mM DTT, 100 μg/ml cycloheximide, 20
  U/ml Superase-IN
  Volumes: 1 gradient = 12 ml total volume (~6 ml 10% sucrose solution, ~6 ml 50% sucrose solution) → For 6 gradients (which can be spun simultaneously in SW41 Ti rotor), 40 ml of each sucrose solution is sufficient

## References

[R1] Arava Y, Wang Y, Storey JD, Liu CL, Brown PO, Herschlag D (2003). Genome-wide analysis of mRNA translation profiles in *Saccharomyces cerevisiae*. Proc Natl Acad Sci U S A.

[R2] Dever TE, Green R (2012). The elongation, termination, and recycling phases of translation in eukaryotes. Cold Spring Harb Perspect Biol.

[R3] Di Santo R, Aboulhouda S, Weinberg DE (2016). The fail-safe mechanism of post-transcriptional silencing of unspliced HAC1 mRNA. Elife.

[R4] Floor SN, Doudna JA (2016). Tunable protein synthesis by transcript isoforms in human cells. Elife.

[R5] Hinnebusch AG, Lorsch JR (2012). The mechanism of eukaryotic translation initiation: new insights and challenges. Cold Spring Harb Perspect Biol.

[R6] Ingolia NT, Ghaemmaghami S, Newman JR, Weissman JS (2009). Genome-wide analysis *in vivo* of translation with nucleotide resolution using ribosome profiling. Science.

[R7] Mašek T, Valasek L, Pospisek M (2011). Polysome analysis and RNA purification from sucrose gradients. Methods Mol Biol.

[R8] Odegaard JI, Lee MW, Sogawa Y, Bertholet AM, Locksley RM, Weinberg DE, Kirichok Y, Deo RC, Chawla A (2016). Perinatal licensing of thermogenesis by IL-33 and ST2. Cell.

[R9] Pfaffl MW (2001). A new mathematical model for relative quantification in real-time RT-PCR. Nucleic Acids Res.

[R10] Warner JR, Knopf PM, Rich A (1963). A multiple ribosomal structure in protein synthesis. Proc Natl Acad Sci U S A.

[R11] Weinberg DE, Shah P, Eichhorn SW, Hussmann JA, Plotkin JB, Bartel DP (2016). Improved ribosome-footprint and mRNA measurements provide insights into dynamics and regulation of yeast translation. Cell Rep.

